# Liquid biopsy in pancreatic ductal adenocarcinoma: current status of circulating tumor cells and circulating tumor DNA

**DOI:** 10.1002/1878-0261.12537

**Published:** 2019-07-30

**Authors:** Jee‐Soo Lee, Sung Sup Park, Young Kyung Lee, Jeffrey A. Norton, Stefanie S. Jeffrey

**Affiliations:** ^1^ Department of Laboratory Medicine Hallym University Sacred Heart Hospital Anyang Korea; ^2^ Department of Laboratory Medicine Seoul National University College of Medicine Seoul Korea; ^3^ Department of Laboratory Medicine Hallym University College of Medicine Anyang Korea; ^4^ Department of Surgery Stanford University School of Medicine Stanford CA USA

**Keywords:** circulating tumor cells, circulating tumor DNA, liquid biopsy, pancreatic cancer, pancreatic ductal adenocarcinoma, tumor‐derived circulating cell‐free DNA

## Abstract

Reliable biomarkers are required to evaluate and manage pancreatic ductal adenocarcinoma. Circulating tumor cells and circulating tumor DNA are shed into blood and can be relatively easily obtained from minimally invasive liquid biopsies for serial assays and characterization, thereby providing a unique potential for early diagnosis, forecasting disease prognosis, and monitoring of therapeutic response. In this review, we provide an overview of current technologies used to detect circulating tumor cells and circulating tumor DNA and describe recent advances regarding the multiple clinical applications of liquid biopsy in pancreatic ductal adenocarcinoma.

**Abbreviations**
AJCC: American Joint Committee on Cancer
ALDH: aldehyde dehydrogenase
ARMS: amplification-refractory mutation system
ASCO: American Society of Clinical Oncology
BD‐IPMN: branch duct type intraductal papillary mucinous neoplasm
BEAMing: beads, emulsion, amplification, and magnetics
BPER: base‐position error rate
CAP: College of American Pathologists
cast‐PCR: competitive allele‐specific TaqMan polymerase chain reaction
CCGA: Circulating Cell Free Genome Atlas
cfDNA: circulating cell‐free DNA
COLD‐PCR: coamplification at lower denaturation temperature polymerase chain reaction
CRP: cancer resistance pathway
CSC: cancer stem cell
CTCs: circulating tumor cells
ctDNA: circulating tumor DNA
CTM: circulating tumor microemboli
DAPI: 4′,6‐diamidino‐2‐phenylindole
ddPCR: droplet digital polymerase chain reaction
DEP: dielectrophoresis
DFS: disease‐free survival
dPCR: digital polymerase chain reaction
EMT: epithelial–mesenchymal transition
EpCAM: epithelial cell adhesion molecule
EPISPOT: Epithelial ImmunoSPOT Assay
ESA: epithelial‐specific antigen
EUS‐FNA: endoscopic ultrasound‐guided fine needle aspiration
EV: extracellular vesicle
FISH: fluorescence in situ hybridization
FMSA: flexible micro spring array
GEDI: geometrically enhanced differential immunocapture
GEM: geometrically enhanced mixing
GO: graphene oxide
GSI: γ‐secretase inhibitor
HB: herringbone
HDAC: histone deacetylase
iDES: integrated digital error suppression
IF: immunofluorescence
IHC: immunohistochemical
IPMN: intraductal papillary mucinous neoplasm
ISET: isolation by size of epithelial tumor cells
LNA‐dPNA PCR clamp: locked nucleic acid-dual peptide nucleic acid polymerase chain reaction clamp
MD‐IPMN: main duct type intraductal papillary mucinous neoplasm
NGS: next‐generation sequencing
OS: overall survival
PARE: personalized analysis of rearranged ends
PB: peripheral blood
PDAC: pancreatic ductal adenocarcinoma
PFS: progression‐free survival
PNA: peptide nucleic acid
PV: portal vein
QMS: quadrupole magnetic sorter
qPCR: quantitative polymerase chain reaction
Safe‐SeqS: safe‐sequencing system
SE‐iFISH: subtraction enrichment and immunostaining-FISH
SLB: supported lipid bilayer

## 1 Pancreatic ductal adenocarcinoma

Pancreatic cancer is the fourth leading cause of cancer mortality in the United States (Kamisawa *et al*., 2016). In 2018, the American Cancer Society estimated that there will be 55 440 newly diagnosed cases and 44 330 deaths from pancreatic cancer (Siegel *et al*., 2018). Approximately 95% of pancreatic cancers are classified as exocrine cancers, while less than 5% of pancreatic cancers are endocrine cancers, namely, pancreatic neuroendocrine tumors. The exocrine cancers include pancreatic adenocarcinoma, acinar cell carcinoma, cystadenocarcinoma, and pancreatoblastoma: Pancreatic adenocarcinoma, or pancreatic ductal adenocarcinoma (PDAC), is the major histological subtype that comprises about 90% of all pancreatic cancers (Goel and Sun, 2015).

The TNM stages of pancreatic cancer are based on American Joint Committee on Cancer (AJCC) Cancer Staging Manual, which consider primary tumor size (T), regional lymph node involvement (N), and distant metastasis (M) (Allen *et al*., 2017; Chun *et al*., 2018; Kamarajah *et al*., 2017; Kamisawa *et al*., 2016). Stages I and II are mostly considered as resectable, and stages III and IV are typically classified as locally advanced and metastatic, respectively. PDACs generally carry a very poor prognosis with the 5‐year survival rate for all stages of PDAC as low as 6–8% (Siegel *et al*., 2018; Ying *et al*., 2016). While surgical resection remains the only curative therapy, less than 20% of patients are candidates for surgical resection, which increases the 5‐year survival rate to 15–25% (Luketina *et al*., 2015; Schlitter *et al*., 2017). Approximately 50–60% of patients are found to have metastasis at diagnosis due to nonspecific or even lack of symptoms that limits earlier diagnosis (Kleeff *et al*., 2016), with only a 3% 5‐year survival for distant disease (Siegel *et al*., 2018).

Clinicians are struggling to develop diagnostic strategies for the early detection of the disease. Adequate biopsy is still challenging because of its poor anatomic location. Endoscopic ultrasound‐guided fine needle aspiration (EUS‐FNA) is preferred for obtaining specimens for biopsy, yet its negative predictive value remains at 16–86% (Mohammad Alizadeh *et al*., 2016). Currently, the serum level of CA 19‐9 is a widely used biomarker for the diagnosis or monitoring of PDAC, but CA19‐9 alone exhibits a wide range of sensitivity (70–95%) and specificity (70–90%) (Ballehaninna and Chamberlain, 2012; Scara *et al*., 2015). False‐negative results are observed in patients with the Lewis‐negative blood group, Le(a‐b‐) that occurring in about 5–10% of Caucasians, and false‐positive results have been reported in other diseases including obstructive jaundice, acute cholangitis, and chronic pancreatitis (Passerini *et al*., 2012; Tanaka *et al*., 2000). Thus, a highly sensitive, reliable, and noninvasive biomarker for evaluating and managing PDAC patients is still required.

## 2 Circulating tumor cells and circulating tumor DNA

Circulating tumor cells (CTCs) and circulating tumor DNA (ctDNA), as liquid biopsies, are an emerging minimally invasive tool for cancer diagnosis, surveillance, and treatment. CTCs can be traced back to their first description by Thomas Ashworth in 1869 (Ashworth, 1869). CTCs are released from primary tumor and/or metastatic sites into the bloodstream. Since CTCs exist as rare cells in the blood (one CTC among 10^6^–10^9^ blood cells), recent studies focus on the efficient capture of rare CTCs from whole blood (Ferreira *et al*., 2016). Investigators use CTCs as a guide to (a) determine prognosis, (b) monitor in real‐time therapeutic responses and tumor recurrence, (c) explore therapeutic targets, and (d) potentially develop new drugs by studying metastatic cancer biology and drug resistance mechanisms in CTCs (Ferreira *et al*., 2016).

ctDNA is a subset of circulating extracellular DNA in plasma (also called cell‐free DNA, cfDNA), specifically released from cancer cells. ctDNA (known as tumor‐derived cfDNA) may originate from apoptotic and necrotic tumor cells, from living tumor cells, or even from CTCs; thus, it has a variable half‐life from 15 minutes up to 2 h (Alix‐Panabieres and Pantel, 2016; Diaz and Bardelli, 2014; Diehl *et al*., 2008; Kidess and Jeffrey, 2013; Nordgard *et al*., 2018). While the size of cfDNA released by apoptotic cells represents approximately 166 bp, ctDNA has recently been reported as being more highly fragmented (Mouliere and Rosenfeld, 2015; Underhill *et al*., 2016). Detecting ctDNA is generally based on the target mutation (e.g., *KRAS*,* BRAF*,* EGFR*, hypermethylation, and multiple gene panels) (Kidess and Jeffrey, 2013). Due to its small fraction (occasionally < 0.01%) among total cfDNA in circulation, approach with sensitive detection methods for ctDNA is highly recommended (Cheng *et al*., 2016). Recent advances in ctDNA analysis highlight future critical roles in cancer management of this easily and serially accessible assay: (a) monitoring tumor burden, (b) evaluating therapeutic response, and (c) identifying therapeutic targets through minimally invasive molecular profiling (Ignatiadis *et al*., 2015). Intratumoral heterogeneity exists due to uneven distribution of cancer subclones in the same tumor (spatial heterogeneity) and due to different genetic alterations that may be selected over time (temporal heterogeneity) as a result of microenvironmental selection, genomic instability, and following multiple drug treatments, where such treatments would ablate cancer cells sensitive to the treatment but not block expansion of residual surviving drug‐resistant cancer cell subpopulations (Dagogo‐Jack and Shaw, 2018; Friedman, 2016; Jeffrey and Toner, 2019; McGranahan and Swanton, 2017). Moreover, most patients with metastatic cancer have multiple rather than solitary metastases, some of which may be discordant with the primary tumor and between other metastases. Sequential tissue sampling of every metastatic lesion is impractical and risky. As a liquid biopsy represents cancer cells or cancer cell products/nucleic acids derived from the entire tumor burdens of the patient, liquid biopsy can be a valuable alternative to tissue biopsies. The following discussion summarizes the current technologies of CTCs and ctDNA and application of these tools to manage patients with PDAC.

## 3 Current technologies in CTCs

Current CTC technologies include two main steps: CTC enrichment and CTC identification. CTC enrichment strategies focus on improving yield of capturing tumor cells, called capture efficiency, and obtaining high‐purity CTCs via depleting the background blood cells (i.e., leukocytes). The most widely used enrichment strategies are based on immunoaffinity, called label dependent, which uses cell surface markers to capture epithelial tumor cells. Immunomagnetic capture is widely used: The specific antibodies are normally conjugated with magnetic nanoparticles, and a magnetic field is then used to capture the CTCs. Tumor‐specific cell surface antigens, such as epithelial cell adhesion molecule (EpCAM), are targeted for the positive enrichment: CellSearch^®^, which is the only US Food and Drug Administration‐approved platform, MACS^®^, and MagSweeper are examples that may use EpCAM‐based or other markers, while AdnaTest uses a cocktail of antibodies against multiple antigens (e.g., EpCAM, EGFR, and HER2). In contrast, negative enrichment is the depletion of nonspecific background cells (i.e., leukocytes) using anti‐CD45 antibodies not expressed by tumor cells: MACS^®^, Quadrupole Magnetic Sorter (QMS), Dynabeads^®^, and EasySep™ are based on this strategy.

Antibodies can also be attached to microposts and other surfaces for CTC capture. Microfluidic devices have been developed based on the technology controlling the fluid flow, which offers advantages for CTC research such as improved capture efficiency and high purity (Warkiani *et al*., 2016). The geometrically enhanced differential immunocapture (GEDI) device uses geometrically enhanced microstructures and combines positive enrichment with hydrodynamic chromatography, which additionally enables cell size‐based separation. Surface‐capture microfluidic devices, such as Herringbone (HB) Chip, Geometrically enhanced mixing (GEM) chip, Graphene oxide (GO) Chip, and the modular sinusoidal system (BioFluidica), increase collision events between the cells and the surface‐coated antibodies. The other kind of microfluidic devices, such as CTC‐iChip, IsoFlux™, LiquidBiopsy, Ephesia chip, and Magnetic Sifter, use microfluidic‐ and immunomagnetic‐based strategies, and these devices exhibited higher sensitivity in CTC separation than CellSearch^®^ (Karabacak *et al*., 2014).

Another major type of CTC enrichment strategies, known as label‐independent enrichments, relies on biophysical properties (e.g., size, including inertial focusing, electrical charge, and density). A substantial number of microfiltration systems are based on the principle that tumor cells (12–25 μm) are basically larger than leukocytes (8–14 μm) (Sollier *et al*., 2014). Therefore, these systems use 7–8 μm pores [isolation by size of epithelial tumor cells (ISET) filter device, ScreenCell^®^, and CellSieve™], or less (VyCAP microsieves which have a membrane thickness smaller than the pore size), microfabricated filter membranes [Flexible Micro Spring Array (FMSA) (Harouaka *et al*., 2014)], or 3‐dimensional microfiltration layers (FaCTChecker, Resettable Cell Trap, and Cluster‐Chip). Inertial focusing microfluidics can be applied for size‐based separations (Vortex and ClearCell^®^ systems). Dielectrophoresis (DEP) uses the polarizabilities of cells in a nonuniform electrical field. In the electrical field, cells are pushed by either negative or positive force and separated based on their cell size and polarizability. Commercialized DEP systems include ApoStream^®^ and DEPArray™. Recently, microfluidic platforms applied both cell size‐ and deformability‐based systems for CTC enrichment: The Parsortix™ (Xu *et al*., 2015, 2017) and Celsee™ (Gogoi *et al*., 2016). A density‐based gradient technology has been also commercialized for separating CTCs: Ficoll‐Paque^®^, RosetteSep™, OncoQuick^®^, and Lymphoprep™. Viable CTCs can be further characterized through combining functional assay with capturing CTCs (Alix‐Panabieres *et al*., 2016). The method that targets secreted tumor‐associated analytes [i.e., Epithelial ImmunoSPOT Assay (EPISPOT)] and the assay based on cell adhesion matrix (CAM) (i.e., Vita‐Assay™ and Vita‐Cap™) are commercially available (references for technology platforms described above are cited in (Ferreira *et al*., 2016).

After enrichment of CTCs, verification of the captured cells is subsequently required. Immunofluorescence (IF) staining, which usually defines 4′,6‐diamidino‐2‐phenylindole (DAPI) + (nuclear stain), CD45− (leukocyte marker), and cytokeratin (CK) + (epithelial marker), which identify epithelial‐like CTCs, is most extensively used, but immunohistochemical (IHC) staining using chromogenic reporters, fluorescence in situ hybridization (FISH), and molecular analyses ranging from reverse transcription polymerase chain reaction (RT–PCR) to aptamer‐based assays to targeted sequencing is also used (Paterlini‐Brechot and Benali, 2007; Smith *et al*., 2007; Swennenhuis *et al*., 2009).

## 4 Clinical application of CTCs in PDAC

Previous CTC studies in pancreatic cancer are summarized in Table 1.

**Table 1:** Previous CTC studies focusing on pancreatic cancer

Enrichment strategy		Refs	N	Stage	Detection strategy		Detection rate	Enumeration
IM CellSearch^®^	EpCAM	Dotan *et al*. (2016)	48	IV	IF	DAPI+/CD45−/panCK+, MUC‐1+	48% (23/48) (≥ 1 CTC): 13% (6/48) (≥ 2 CTCs); 8% (3/37) (≥ 2 CTCs) at first evaluation after Tx	NA
		Piegeler *et al*. (2016)	8	IB (*n* = 1) IIB (*n* = 1) III (*n* = 2) IV (*n* = 4)	IF	DAPI+/CD45−/CK+	87.5% (7/8): 100% (1/1) in Stage IB; 100% (1/1) in Stage IIB; 100% (2/2) in Stage III; 75% (3/4) in Stage IV	Median 4.5 CTCs/7.5 mL, range 0–83 CTCs/7.5 mL
		Bissolati *et al*. (2015)	20	R	IF	DAPI+/CD45−/panCK+	20% (4/20) in PB 40% (8/20) in PV	NA
		Catenacci *et al*. (2015)	14	IIB‐IV	IF	DAPI+/CD45−/EpCAM+	21.4% (3/14) in PB 100% (14/14) in PV	(1) In PB, mean 0.7 CTCs/7.5 mL, median 0 CTCs/7.5 mL, range 0–7 CTCs/7.5 mL. (2) In PV, mean 125.64 CTCs/7.5 mL, median 68.5 CTCs/7.5 mL, range 1–516 CTCs/7.5 mL
		Earl *et al*. (2015)	35	R (*n* = 10) LA (*n* = 11) M (*n* = 14)	IF	DAPI+/CD45−/CK+	20% (7/35) in total 10% (1/10) in R 42.8% (6/14) in M	Mean 0.77 CTCs/7.5 mL in total, mean 0.1 CTCs/7.5 mL in R, mean 1.9 CTCs/7.5 mL in M
		Bidard *et al*. (2013)	79	III	IF	CD45−/CK+, EGFR	5% (4/75) at baseline 9% (5/56) at first evaluation 11% (9/79) in total 50% (2/4) in Stage IV (control)	1–15 CTCs/7.5 mL
		Kurihara *et al*. (2008)	26^a^	II (*n* = 1) III (*n* = 1) IVA (*n* = 10) IVB (*n* = 14)	IF	DAPI+/CD45−/panCK+	42% (11/26) in total 45.8% (11/24) in Stage IV	Mean 16.9 CTCs/7.5 mL, range 1–105 CTCs/7.5 mL
		Allard *et al*. (2004)	16^a^	IV	IF	DAPI+/CD45−/panCK+	19% (4/21) (≥ 2 CTCs) of the samples	Mean 2 ± 6 CTCs/7.5 mL, median 3.5 CTCs/7.5 mL
IM CellCollector^®^	EpCAM	El‐Heliebi *et al*. (2018)	15	I (*n* = 7) II‐III (*n* = 6) NA (*n* = 2)	RCA, IF	*KRAS,* DAPI+/CD45−/CK18 or PanCK±	47% (7/15) *KRAS* ^mut^ 40% (6/15)	Range 1–3 CTCs/patient, *KRAS* ^mut^ 1–8 RCPs/CTC, *KRAS* ^wt^ 1–2 RCPs/CTC
IM MACS	EpCAM	Effenberger *et al*. (2018)	69	I (*n* = 2) II (*n* = 30) III (*n* = 10) IV (*n* = 27)	IF	DAPI+/CD45−/CK+	33.3% (23/69)	Range 1–19 CTCs/7.5 mL
		Zhou *et al*. (2011)	25^a^	I‐II (*n* = 5) III (*n* = 8) IV (*n* = 12)	RT–PCR	h‐TERT, CK20, CEA, C‐MET	100% (25/25)	NA
IM Dynabeads^®^	MUC1 EpCAM	de Albuquerque *et al*. (2012)	34	II‐IV	RT–PCR	KRT19, MUC1, EPCAM, CEACAM5, BIRC5	47.1% (16/34): 20.6% for KRT19 and MUC1; 23.5% for EPCAM; 2.9% for CEACAM5; 17.6% for BIRC5	NA
IM	anti‐cMET	Zhang *et al*. (2016b)	7	NA	IF, FISH	DAPI+/CD45−/c‐MET+, MET FISH	0% with c‐MET CTC assay 14% (1/7) with CellSearch^®^	Range 0–1 CTCs/7.5 mL (CellSearch^®^)
IM SE	CD45(−)	Zhang *et al*. (2015b)	22^a^	I (*n* = 2) II (*n* = 10) III (*n* = 4) IV (*n* = 6)	IF, FISH	DAPI+/CD45−/CK+ and/or CEP8 signal number > 2, DAPI+/CD45−/CK− and CEP8 signal number > 2	68.2% (15/22) (≥ 2 CTCs) in total: 9.1% (2/22) with CK+; 59.1% (13/22) with CK−; 9.1% (2/22) (> 10 CTCs); 78.6% (11/14) (≥ 2 CTCs) in PDAC	Median 3 CTCs/3.5 mL, range 0–60 CTCs/3.5 mL, 60 CTCs/3.5 mL in a Pt with stage II, 14 CTCs/3.5 mL in a Pt with stage IV
IM SE	CD45 (−)	Wu *et al*. (2018)	19	IIA (*n* = 3) IIB (*n* = 11) III (*n* = 4) IV (*n* = 1)	IF, FISH	DAPI+/CD45−/CK+ and/or CEP8 signal number > 2	26.3% (5/19) CTMs in total: 21.1% (4/19) at baseline; 27.3% (3/11) in stage IIB; 25% (1/4) in stage III; 100% (1/1) in stage IV	Median 5 CTCs/7.5 mL (at baseline), range 1–30 CTCs/7.5 mL (at baseline)
IM SE	CD45 (−)	Gao *et al*. (2016)	25	I (*n* = 5) II (*n* = 8) III (*n* = 6) IV (*n* = 6)	IF, FISH	DAPI+/CD45−/CK18 + or CEP8 signal number > 2	88% (22/25)	Median 3 CTCs/7.5 mL, range 0–13 CTCs/7.5 mL
IM MACS	CD45 (−)	Zhang *et al*. (2015a)	13	NA	IF, Aptamer, FISH	DAPI+/CD45−/panCK+, DAPI+/CD45−/BC‐15+	84.6% (11/13)	Mean 34.4 CTCs/7.5 mL (panCK+), mean 24 CTCs/7.5 mL (BC‐15 + )
		Ren *et al*. (2011)	41	III‐IV	IF	DAPI+/CA19‐9 + /CK+	80.5% (33/41) (≥ 2 CTCs)	Mean 16.8 ± 16.0 CTCs/7.5 mL, range 0–59 CTCs/7.5 mL
SLB, μF CMx chip	EpCAM	Chang *et al*. (2016)	63	I (*n* = 1) II (*n* = 32) III (*n* = 10) IV (*n* = 20)	IF	DAPI+/CD45−/panCK+	81% (51/63) CTCs 81% (51/63) CTMs (multiple cells ≥ 2 CTCs)	Mean 70.2 CTCs/2 mL, mean 29.5 CTMs/2 mL
		Tien *et al*. (2016)	41	IA‐III	IF	DAPI+/CD45−/panCK+	39% (16/41) in PB 58.5% (24/41) in PV	(1) In PB, mean CTCs 92.0/2 mL, median CTCs 52.0/2 mL. (2) In PV, mean CTCs 313.4/2 mL, median CTCs 116.5/2 mL
IM, μF Parallel flow micro aperture chip	EpCAM anti‐CEA Size‐based filtration	Chang *et al*. (2015)	12^a^	IV	IF	DAPI+/CD45−/CK+	91.7% (11/12)	Mean 26 ± 11 CTCs/8 mL, range 0–42 CTCs/8 mL, mean 31 CTCs/8 mL (untreated Pts), mean 22 CTCs/8 mL (treated Pts)
μF Nanostructured capture NanoVelcro chip	EpCAM	Court *et al*. (2018)	100	I (*n* = 9) II (*n* = 31) III (*n* = 31) IV (*n* = 29)	IF	DAPI+/CD45−/CK+	78% (78/100): 44.4% (4/9) in stage I; 74.2% (23/31) in stage II; 77.4% (24/31) in stage III; 93.1% (27/29) in stage IV	Median 2 (IQR 1–6) CTCs/4 mL in total, median 7 (IQR 3–13) CTCs/4 mL in occult metastatic Pts
μF Micropost GEDI	Size‐based filtration EpCAM	Rhim *et al*. (2014)	11	I (*n* = 1) IIA (*n* = 1) IIB (*n* = 1) III (*n* = 1) IV (*n* = 7)	IF	DAPI+/CD45−, DAPI+/CD45−/CK+	73% (8/11) in PDAC 40% (8/21) in Cystic lesion	Mean 14.1 ± 18.1 CTCs/mL (PDAC), mean 4.5 ± 7.3 CTCs/mL (Cystic lesion)
μF Cell surface capture GEM	EpCAM	Sheng *et al*. (2014)	18^a^	IV	IF	DAPI+/CD45−/CK+	94.4% (17/18)	Range 0–23 CTCs/7.5 mL
μF Cell surface capture BioFluidica	EpCAM	Kamande *et al*. (2013)	12	R (*n* = 5) M (*n* = 7)	IF	DAPI+/CD45−/EpCAM+	100% (7/7) in M	Mean 53CTCs/mL in M, median 51 CTCs/mL in M, range 9–95 CTCs/mL in M, mean 11 CTCs/mL in R
μF Cell surface capture Slit filtrationeDAR	EpCAM Size‐based filtration	Zhao *et al*. (2013)	10^a^	IV	IF	Hoechst+/CD45 + /EpCAM+/CK+	80% (8/10)	Range 2–872 CTCs/mL
Size‐based filtration ISET	Pore size 8.0 μm	Poruk *et al*. (2016)	50	I (*n* = 8) II (*n* = 38)IV (*n* = 4)	IF	DAPI+/CD45−/panCK+, DAPI+/CD45−/vimentin+	78% (39/50) with eCTCs 52% (26/50) with mCTCs	Median 30 eCTCs/mL, range 1–251 eCTCs/mL, median 3 mCTCs/mL, range 1–16 mCTCs/mL
		Khoja *et al*. (2012)	53	M or Inoperable	Light microscope, IHC	CD45−, Morphology	88.9% (24/27) (ISET) 39.6% (21/53) (CellSearch^®^)	Mean 26 CTCs/7.5 mL (ISET), median 9 CTCs/7.5 mL (ISET), range 0–240 CTCs/7.5 mL (ISET), mean 2 CTCs/7.5 mL (CellSearch^®^), median 0 CTCs/7.5 mL (CellSearch^®^) range 0–15 CTCs/7.5 mL (CellSearch^®^)
Size‐based filtration ScreenCell	Pore size 7.5 μm	Sefrioui *et al*. (2017)	58^a^	L (*n* = 16) LA (*n* = 18) M (*n* = 24)	Light microscope	Morphology	56% (33/49) in available samples: 57% (16/28) in L‐LA; 81% (17/21) in M	Median 1 CTC/mL, range 0–151 CTCs/mL
		Kulemann *et al*. (2016)	21	IIA (*n* = 2) IIB (*n* = 8) III (*n* = 4) IV (*n* = 7)	IF, Light microscope, IHC, PCR	Hoechst+/CK+, Hoechst+/ZEB‐1 + , Morphology, *KRAS*	86% (18/21) including *KRAS* ^mut^: 100% (2/2) in Stage IIA; 75% (6/8) in Stage IIB; 75% (3/4) in Stage III; 100% (7/7) in Stage IV 66.7% (14/21) with cytology only 23.8% (5/21) CTC clusters 57.1% (4/7) ZEB1 + in Stage IV	Mean 0.5 CTCs/3 mL, range 0–37 CTC/3 mL
		Cauley *et al*. (2015)	105	IA‐IV	Light microscope	Morphology	49% (51/105)	NA
		Kulemann *et al*. (2015)	11	IIB (*n* = 4) III (*n* = 3) IV (*n* = 4)	Light microscope, RT–PCR	Morphology, *KRAS*	18% (2/11) with cytology 73% (8/11) with *KRAS* ^mut^: 75% (3/4) in Stage IIB; 100% (3/3) in Stage III; 50% (2/4) in Stage IV	NA
		Iwanicki‐Caron *et al*. (2013)	27	R (*n* = 9) LA (*n* = 9) M (*n* = 9)	Light microscope	Morphology	55.6% (15/27) in total: 44.4% (4/9) in R; 66.7% (6/9) in LA; 55.6% (5/9) in M	NA
Size‐based filtration FMSA (vs. CellSearch_®_)	Microfiltration	Ma *et al*. (2015)	2^a^	IIB (*n* = 1) IV (*n* = 1)	Ad5GTSe infection/GFP, IF	GFP+, CK+/CD45−	100% (2/2) (FMSA), 50% (1/2) (CellSearch^®^)	13–30 CTCs/7.5 mL (FMSA), 0–1 CTCs/7.5 mL (CellSearch^®^)
Size‐based filtration MetaCell	Pore size 8.0 μm	Bobek *et al*. (2014)	17	I (*n* = 1) IIA (*n* = 4) IIB (*n* = 4) III (*n* = 5) IV (*n* = 3)	IF, Light microscope, IHC	DAPI+/CK18 + , Morphology, MGS, CK, CEA, Vimentin	76.5% (13/17) in total: 78.6% (11/14) in Stage I‐III; 66.7% (2/3) in Stage IV	NA
Density Gradient Ficoll‐Paqueplus	Density Gradient	Gorner *et al*. (2015)	6	II (*n* = 2) III (*n* = 1) IV (*n* = 3)	FACS RT–PCR	Hoechst+/CD45−/EpCAM+, Integrin+, or MUC+, c‐MET, AGR2, EpCAM, Krt‐19, CD45	66.6% (4/6): 66.6% (2/3) in Stage II‐III; 66.6% (2/3) in Stage IV	NA
CAM assay		Premasekharan *et al*. (2016)	2	IV	FACS	DAPI+/CD45−/CAM^high^/CD14^low^	100% (2/2)	NA
oHSV1‐hTERT‐GFP	Telomerase RT positive cancer cells GFP+ (viable cells)	Zhang *et al*. (2016a)	17	IIB (*n* = 1) III (*n* = 4) IV (*n* = 12)	IF FACS	CD45−/GFP+	88.2% (15/17)	Mean 43.1 CTCs/4 mL
No enrichment		Marrinucci *et al*. (2012)	18^a^	IV	IF	DAPI+/CD45−/CK+	61% (11/18) (≥ 2 CTCs), 50% (9/18) (≥ 5 CTCs)	Mean 15.8 CTCs/mL

CAM, cell adhesion matrix; CTC, circulating tumor cell; CTM, Circulating tumor microemboli; eCTC, epithelial‐like CTC; FISH, fluorescent in situ hybridization; IF, immunofluorescence; IHC, immunohistochemistry; IM, immunomagnetic; IQR, interquartile range; LA, locally advanced; M, metastatic; mCTC, mesenchymal‐like CTC; *N*, number of patients; NA, not available; PB, peripheral blood; PDAC, pancreatic ductal adenocarcinoma; PV, portal vein; R, resectable; RCA, rolling‐circle amplification using padlock probe; RCP, rolling‐circle product; Refs, references; SE, subtraction enrichment; SLB, supported lipid bilayer; Tx, treatment; Pt, patient; μF, microfluidic.

a Various tumor types of pancreatic cancers are included.

### 4.1 Detection

The detection of CTCs in patients of pancreatic cancer has been compared with that in patients with other cancers in previous studies. Using the CellSearch^®^ system, *Allard et al*. enumerated CTCs in 2183 blood samples from 946 metastatic patients with 12 different cancer types, which included 21 blood samples from 16 patients with pancreatic cancer. Lower number of CTCs was detected in pancreatic cancer (mean, 2 CTCs/7.5 mL) than any other carcinomas, such as prostate cancer, ovarian cancer, breast cancer, gastric cancer, colorectal cancer, bladder cancer, rental cancer, and lung cancer. CTCs above the cutoff level (≥2 CTCs) were detected in only 4 out of 21 samples (19%) (Allard *et al*., 2004).

In contrast, recent works using state‐of‐the‐art techniques demonstrated comparable detection rates of CTCs in pancreatic cancer when compared with those in different types of carcinomas. Zhang *et al*. (2016a) used hTERT promoter‐regulated oncolytic herpes simplex virus‐1 that targets telomerase reverse transcriptase‐positive tumor cells, and identified CTCs in 88.2% (15/17) of patients with various stages of pancreatic cancer. *Chang et al*. developed a parallel flow microfluidic chip that is combined with different strategies such as immunomagnetics and size‐based filtration. This device performed well for isolating of CTCs in patients with metastatic pancreatic cancer (91.7%, 11/12 in pancreatic cancer; 100%, 38/38 in non‐small‐cell lung cancer) (Chang *et al*., 2015). Another study by *Ting et al*. applied the microfluidic CTC‐iChip, which depletes normal blood cells by inertial focusing size‐based sorting and separates CTCs immunomagnetically, for single‐cell RNA sequencing. In this study, median 118 CTCs/mL (range, 0–1694) were detected in pancreatic tumor‐bearing mice (KPC mice) (Ting *et al*., 2014). Varillas *et al*. (2017) have introduced a detailed procedure for using a microfluidic chip with a herringbone structure and reported that this device could consistently detect a low number of CTCs in pancreatic cancer. Interestingly, *El‐Heliebi et al*. applied *KRAS* as a marker for CTC enumeration and molecular characterization. They used an *in vivo* isolation of CTCs (GILUPI CellCollector^®^) directly from the vein of patients and applied signal amplification of in situ padlock probes via rolling‐circle amplification: 47% (7/15) of patients were CTC‐positive (range, 1–3 CTCs/patient), and 40% (6/15) of patients had *KRAS* mutant CTCs (El‐Heliebi *et al*., 2018).

With regard to the enrichment strategies, size‐based filtering strategies exhibited higher sensitivity in isolating CTCs compared with EpCAM‐based approaches in patients with metastatic or inoperable pancreatic cancer: ISET and CellSearch^®^ detected CTCs in 88.9% (38/50) and in 39.6% (21/53) of patients, respectively (Khoja *et al*., 2012). A recent study by *Brychta et al*. compared the performance of these two strategies by cell spiking experiments [EpCAM‐based CTC isolation (IsoFlux) *vs*. automated size‐based filtration (Siemens Healthineers)]: Especially for low EpCAM expressing cells, the filtration‐based strategy exhibited higher recovery rate (52%) than the IsoFlux device (1%). Additional experiments using the filtration‐based strategy were able to capture CTCs in 42% of frozen diagnostic leukapheresis (DLA) samples from 19 patients with pancreatic cancer. Although there was no difference in prevalence of CTCs in samples from patients with and without metastases (44% vs 40%, respectively), CTC numbers were somewhat higher when distant metastases were present (0–7 for Stage IV disease versus 0–2 for stages 2b‐III) (Brychta *et al*., 2017).

### 4.2 Early diagnosis

The potential role of CTCs as an early diagnostic marker has recently been revealed by *Rhim et al*. (Table 2). Using GEDI chip, CTCs were captured in three different subject groups [PDAC patients at all stages, patients with precancerous cystic lesion, that is, intraductal papillary mucinous neoplasm (IPMN) or mucinous cystic neoplasm, and cancer‐free controls]. Interestingly, CTCs were detected in 40% (8/21) of the patients with precancerous lesions: Circulating pancreas epithelial cells may precede the detectable tumors. The detection rates of CTCs were 73% (8/11) and 0% (0/19) in PDAC patients and cancer‐free group, respectively (Rhim *et al*., 2014).

**Table 2:** Studies investigating the role of CTC/ctDNA detection in early cancer diagnosis

References	Patients	Analyte	Methods	Results	Comments
Rhim *et al*. (2014)	PDAC (*n* = 11), Precancerous cystic lesions (*n* = 21): Side‐branch IPMN (*n* = 18); MCN (*n* = 3) Cancer‐free controls (*n* = 19)	CTC	microfluidic platform GEDI	CTCs were captured in: 8 of 11 (73%) patients with PDAC 8 of 21 (40%) patients with cystic lesions; 0 of 19 (0%) cancer‐free controls	Pancreas epithelial cells can be detected in patients with cystic lesions of pancreas before the clinical diagnosis of cancer.
Berger *et al*. (2016)	PDAC (stage IV) (*n* = 24), IPMN (*n* = 21), Borderline IPMN (*n* = 16), SCA (*n* = 26), Cancer‐free controls (*n* = 38)	ctDNA	ddPCR (Bio‐Rad)	mean cfDNA value of: 4.220 ± 2.501 ng·µL^−1^ in PDAC; 0.2887 ± 0.0319 ng·µL^−1^ in IPMN; 0.1360 ± 0.0203 ng·µL^−1^ in controls, *GNAS* ^mut^ ctDNA: 6 of 24 (25.0%) with PDAC; 15 of 21 (71.4%) with IPMN; 0% with SCA and controls. *KRAS* ^mut^ ctDNA: 10 of 24 (41.7%) with PDAC; 0% with IPMN, SCA and controls	cfDNA discriminates IPMN patients from controls Detection of *GNAS* and *KRAS* mutations discriminates IPMN patients from those with harmless pancreatic tumors

cfDNA, cell‐free DNA; CTC, circulating tumor cell; ctDNA, circulating tumor DNA; ddPCR, droplet digital PCR; IPMN, intraductal papillary mucinous neoplasm; MCN, mucinous cystic neoplasm; PDAC, pancreatic ductal adenocarcinoma; SCA, serous cystadenoma.

### 4.3 A marker of advanced disease

The correlation of CTC levels with more aggressive pathologic features and with advanced disease is still debated. A multicenter randomized clinical trial suggested that CTC detection with CellSearch^®^ significantly correlated with aggressive tumor differentiation (Bidard *et al*., 2013). In another study, which used a modular microfluidic system, CTC levels isolated from metastatic PDAC patients (mean 53 CTCs/mL, *n* = 7 patients) was significantly higher than those from resectable PDAC patients (mean 11 CTCs/mL, *n* = 5 patients), although further testing will be required because of the small numbers of patients tested in this first proof‐of‐principle assay (Kamande *et al*., 2013). The expression of *C‐MET*,* CK20*, and *CEA* mRNA detected by RT–PCR after MACS purification correlated with TNM stage (Zhou *et al*., 2011). More recently, Court *et al*. (2018) preoperatively enumerated CTC using the microfluidic NanoVelcro chip and reported that PDAC patients with occult metastatic disease had significantly more CTCs than PDAC patients with localized disease (median 7 CTCs *vs*. 1 CTC, *P *<* *0.0001).

In contrast, Cauley *et al*. (2015) described that CTC positivity was not associated with tumor characteristics, lymph node metastasis, respectability, and advanced TNM stage. Similarly, the percentage of CTC detection using size‐based filtration was not associated with the TNM stage or distant metastasis (Bobek *et al*., 2014; Kulemann *et al*., 2015).

### 4.4 Prognosis

Studies investigating the role of CTC detection as a prognostic marker are summarized in Table 3. Research efforts on CTC enumeration for better prognostic classification are well underway. Several studies discussed below performed multivariable analysis using the Cox regression model, which exhibits CTCs as an independent prognostic factor. Bidard *et al*. (2013) conducted multicenter randomized clinical trial evaluating 79 patients with locally advanced nonmetastatic PDAC. Patients were randomly assigned to receive gemcitabine alone, or gemcitabine plus erlotinib. The CTC positivity was measured by CellSearch^®^ at two different time points (at baseline and at two months): The overall detection rate of CTCs (either at baseline or at two months) was 11%. CTC positivity in locally advanced pancreatic adenocarcinoma at any time point was an independent prognostic factor for overall survival (OS) in multivariable analysis but not for progression‐free survival (PFS). A more recent study by *Effenberger et al*. enrolled 69 patients with PDAC and identified CTCs using MACS enrichment: Here, CTC positivity was an independent risk factor of reduced PFS (HR = 4.543, *P *=* *0.006) and OS (HR = 2.093, *P *=* *0.028) (Effenberger *et al*., 2018). Studies using different platforms in PDAC patients exhibited association of CTCs with survival rates. *Chang et al*. used a supported lipid bilayer (SLB) surface‐coated microfluidic chip (CMx platform): Patients with unfavorable circulating tumor microemboli (CTM) levels exhibited shorter PFS and OS when compared with patients with favorable CTM levels (PFS, 2.7 months *vs*. 12.1 months, *P *<* *0.0001; OS, 6.4 months *vs*. 19.8 months, *P *<* *0.0001). These associations were still observed in each subgroup (early stage and advanced stage) (Chang *et al*., 2016). Gao *et al*. (2016) applied EpCAM independent subtraction enrichment and immunostaining‐FISH (SE‐iFISH) to enumerate CTCs and demonstrated that the presence of ≥ 3 CTCs/7.5 mL was the strong predictive factor for worse OS (HR = 4.547, *P *=* *0.016). *Poruk et al*. compared epithelial CTCs and mesenchymal‐like CTCs using IF staining for panCK and vimentin markers, respectively, after the size‐based CTC separation. The epithelial CTCs (CK‐positive) were strongly associated with poorer survival but not mesenchymal‐like CTCs (*P *<* *0.01 vs. *P *=* *0.39). With regard to median time to recurrence, detection of CTCs expressing both CK and vimentin was the significant predictive factor for earlier recurrence (*P *=* *0.01) (Poruk *et al*., 2016). A recent useful meta‐analysis described that detectable baseline CTCs including disseminated tumor cells in the bone marrow was associated with worse disease‐free survival (DFS)/PFS (HR = 1.93, *P *=* *0.007) and OS in pancreatic cancer (HR = 1.84, *P *≤* *0.0001) (Stephenson *et al*., 2017).

**Table 3:** Studies investigating the role of CTC/ctDNA detection as a prognostic marker

References	*N*	Analyte	Methods	Sampling points at	Results
Wu *et al*. (2018)	19	CTC	SET‐iFISH	Before the start of Tx, 10 days after Op, 1 month after Op, 3 months after Op, 7 months after Op	The median OS of the CTM (+) and CTM (−) patients (at baseline) were 7.3 and 25.4 months (*P* = 0.001). The median DFS of the CTM (+) and CTM (−) patients (at baseline) were 1.8 and 18.97 months (*P* = 0.037 )
Court *et al*. (2018)	100	CTC	NanoVelcro chip	Before the start of Tx	CTC positivity was a multivariate predictor of OS (HR, 1.38, *P* = 0.040). CTC count was a univariate predictor of recurrence‐free survival (HR, 2.36, *P* = 0.017).
Effenberger *et al*. (2018)	69	CTC	MACS	Before the start of Tx	CTC positivity was independent risk factor of reduced PFS (HR, 4.543, *P* = 0.006). CTC positivity was independent risk factor of shortened OS (HR, 2.093, *P* = 0.028).
Gao *et al*. (2016)	25	CTC	SE‐iFISH	Before the start of Tx	The median OS of the CTC ≥ 3 and CTC < 3 patients were 10.2 and 15.2 months (*P* = 0.023)
Chang *et al*. (2016)	63	CTC	SLB μF CMx	Before the start of Tx	Survival difference between favorable (CTM < 30) patients and unfavorable (CTM ≥ 30) patients (PFS, 12.1 vs. 2.7 months; OS, 19.8 vs. 6.4 months)
Poruk *et al*. (2016)	50	CTC	ISET	Before the start of Tx	Epithelial CTC positivity was associated with worse survival rate (median survival, 13.7 months vs. not reached, *P* = 0.008)
Zhang *et al*. (2015b)	22	CTC	SE‐iFISH	Before the start of Tx	CTC positivity (≥2/3.75 mL) correlated with worse survival rate (*P* = 0.0458)
Bidard *et al*. (2013)	79	CTC	CellSearch^®^	Before the start of Tx. After 2 months of Tx	CTC positivity (at baseline and/or at 2 months) correlated with poor OS (RR = 2.5, *P* = 0.01)
de Albuquerque *et al*. (2012)	34	CTC	Dynabeads^®^	Before the start of Tx	The median PFS of the CTC (+) and CTC (−) patients were 66.0 and 138.0 days (*P* < 0.01)
Kurihara *et al*. (2008)	26	CTC	CellSearch^®^	Before the start of Tx	The MSTs of the CTC (+) and CTC (−) patients were 110.5 and 375.8 days (*P* < 0.001)
Bernard *et al*. (2019)	194	ctDNA	ddPCR (Bio‐Rad)	Before the start of Tx (*n* = 175): Serially monitored during Tx (*n* = 68)	Baseline ctDNA (+) was associated with shorter PFS (HR = 1.8, *P* = 0.019) in metastatic PDAC. Baseline ctDNA (+) was associated with shorter OS (HR = 2.8, *P* = 0.0045) in metastatic PDAC. Baseline ctDNA and exoDNA MAF ≥ 5% was a significant predictor of OS (HR = 7.73, *P* = 0.00002) in metastatic PDAC
Perets *et al*. (2018)	17	ctDNA	Targeted sequencing (Ion PGM™)	Before the start of Tx	The OS of *KRAS* ^mut^ ctDNA(+) and ctDNA(−) patients were 8 and 37.5 months (*P* < 0.004). The OS negatively correlated with the change in ctDNA levels (between each pair of consecutive samples) (*r* = ‐0.76, *P* = 0.03).
Kim *et al*. (2018)	106	ctDNA	ddPCR (Bio‐Rad)	Before the start of Tx: Every 3 months after Tx	Baseline *KRAS* mutation concentration (HR = 2.08, *P* = 0.009) and *KRAS* fraction (HR = 1.73, *P* = 0.042) were significant prognostic factors for PFS. Baseline *KRAS* mutation concentration (HR = 1.97, *P* = 0.034) was a significant prognostic factor for OS. Increase of cfDNA concentration, *KRAS* ^mut^ ctDNA concentration and *KRAS* fraction (in the sample collected at 6 months after Tx) were correlated with OS (*P* < 0.001, *P* = 0.013, and *P* = 0.036, respectively).
Cheng *et al*. (2017)	188	ctDNA	Targeted sequencing (Hi‐Seq 2500), ddPCR (Bio‐Rad)	Before the start of Tx: For a subset of cases, multiple time points after Tx	*ERBB2* exon 17 mutation (HR = 1.61, *P* = 0.035) and *KRAS* G12V mutation (HR = 1.45, *P* = 0.019) were independent prognostic factors for OS.
Adamo *et al*. (2017)	26	ctDNA	Targeted sequencing (Ion PGM^TM^), ddPCR (Bio‐Rad)	Before the start of Tx	The *KRAS* ^mut^ ctDNA correlated with poorer disease‐specific survival (*P* = 0.018).
Del Re *et al*. (2017)	27	ctDNA	ddPCR (Bio‐Rad)	Before the start of Tx: Subsequently after 15 days of Tx and at first radiologic evaluation	Increase of ctDNA (in the sample collected at day 15) is correlated with PFS and OS (PFS, 2.5 vs 7.5 months, *P* = 0.03; OS 6.5 vs 11.5 months, *P* = 0.009). Baseline *KRAS* ^mut^ was not associated with PFS and OS (*P* = 0.24 and *P* = 0.16).
Pietrasz *et al*. (2017)	135	ctDNA	Targeted sequencing (Ion Proton™) digital PCR (RainDrop™)	Before the start of adjuvant CTx, (*n* = 31). Before the start of Tx (*n* = 104)	The DFS of ctDNA (+) and ctDNA (−) patients were 4.6 and 17.6 months (*P* = 0.03) in resectable PDAC (*n* = 31). The OS of ctDNA(+) and ctDNA(−) patients were 19.3 and 32.2 months (*P* = 0.027) in resectable PDAC (*n* = 31). The OS of ctDNA(+) and ctDNA(−) patients were 6.5 and 19.0 months (*P* < 0.001) in advanced PDAC (*n* = 104 )
Pishvaian *et al*. (2017)	34	ctDNA	Targeted sequencing (Hi‐Seq 2500)	Not mentioned	Detectable ctDNA correlated with poorer OS (*P* = 0.045).
Sefrioui *et al*. (2017)	68	ctDNA	ddPCR (Bio‐Rad)	Before the start of Tx	The median OS of *KRAS* ^mut^ ctDNA(+) and ctDNA(−) patients were 5.2 and 11 months (*P* = 0.01)
Hadano *et al*. (2016)	105	ctDNA	ddPCR (Bio‐Rad)	Before the start of Tx	The DFS of ctDNA (+) and ctDNA (−) patients were 6.1 and 16.1 months (*P* < 0.001). The OS of ctDNA (+) and ctDNA (−) patients were 13.6 and 27.6 months (*P* < 0.0001)
Earl *et al*. (2015)	31	ctDNA	ddPCR (Bio‐Rad)	Before the start of Tx (*n* = 24). After the start of Tx (*n* = 7)	The OS of *KRAS* ^mut^ ctDNA(+) and ctDNA(−) patients were 60 and 772 days (*P* = 0.001).
Kinugasa *et al*. (2015)	75^a^	ctDNA	ddPCR (Bio‐Rad)	Before the start of Tx	The MST of *KRAS* ^mut^ ctDNA(+) and ctDNA(−) patients were 276 and 413 days (*P* = 0.02) – *KRAS* G12V mutation was most well correlated (219 days vs. 410 days)
Sausen *et al*. (2015)	51	ctDNA	ddPCR (Bio‐Rad)	Before the start of Tx: For a subset of cases, multiple time points after surgery	The PFS of ctDNA (+) and ctDNA (−) patients (at baseline) were 7.9 and 15.2 months (*P* = 0.0151). The PFS of ctDNA (+) and ctDNA (−) patients (after surgery) were 9.9 months and not reached (*P* = 0.0199).
Tjensvoll *et al*. (2016)	14^a^	ctDNA	PNA‐mediated real‐time PCR clamping	Before the start of Tx: Subsequently every month during Tx	ctDNA shows trends toward reduced PFS and OS (*P* = 0.064 and 0.066). ctDNA levels before initiation of Tx is independent prognostic factor for PFS and OS (HR 1.31, *P* = 0.047).
Takai *et al*. (2015)	259	ctDNA	digital PCR (RainDrop™)	Before the start of Tx	The *KRAS* ^mut^ ctDNA correlated with poorer OS (*P* < 0.0001).
Singh *et al*. (2015)	127^a^, ^b^	ctDNA	Nested PCR	Not mentioned	The median OS of high cfDNA and low cfDNA patients were 3 and 11 months (*P* = 0.002). *KRAS* ^mut^ was not associated with survival pattern of patients (*P* = 0.398).
Chen *et al*. (2010)	91	ctDNA	Direct sequencing	Before the start of Tx	The MST of *KRAS* ^mut^ ctDNA(+) and ctDNA(−) patients were 3.9 and 10.2 months (*P* < 0.001)

CTC, circulating tumor cell; ctDNA, circulating tumor DNA; CTM, circulating tumor microemboli; CTx, chemotherapy; ddPCR, droplet digital PCR; DFS, disease‐free survival; exoDNA, exosome DNA; MAF, mutant allele fraction; MST, median survival time; *N*, number of patients; Op, operation; OS, overall survival; PFS, progression‐free survival; Tx, treatment.

a ^^Various tumor types of pancreatic cancers are included

b ^^*KRAS* mutation test was available for 110 samples.

### 4.5 Different sampling sites

Research comparing CTCs in portal vein (PV) and those in peripheral blood (PB) is in progress (Table 4). Bissolati *et al*. evaluated PV samplings in 20 patients with nonmetastatic PDAC undergoing surgical resection. Five out of nine CTC‐positive patients had CTCs in PV but not in systemic circulation, detected by CellSearch^®^. At 3‐year follow‐up, patients with detectable CTCs in PV exhibited higher rate of liver metastasis than patients without detectable CTCs in PV (53% *vs*. 8%, *P *=* *0.038) (Bissolati *et al*., 2015). Catenacci *et al*. evaluated CTCs in EUS‐guided PV sampling. Using CellSearch^®^, they detected CTCs in PV blood samples from 100% (18/18) of patients, while only four patients (22.2%) had CTCs in the PB. Even in patients with nonmetastatic and localized or borderline‐resectable pancreatic cancer, high levels of CTCs were detected (mean 83.2 CTCs/7.5 mL) in PV (Catenacci *et al*., 2015). Further recently, Tien *et al*. (2016) collected intraoperative PB and PV samples from 41 PDAC patients. CTC count (CMx platform) in PV was a strong predictor for liver metastasis in a 6‐month follow‐up after surgery (*P *=* *0.002). The PV is the main entrance for distant metastasis of PDAC, and tumor cells spread into blood circulation before radiologically detected. CTCs in PV seem to more closely reflect the metastatic potential, although prospective studies with large cohorts are still required.

**Table 4:** Studies investigating the role of CTCs detected in portal vein samples

References	*N*	Methods	Sampling points at	Results
Bissolati *et al*. (2015)	20	CellSearch^®^	At surgery, before any manipulation of cancer	Liver metastases occurred more frequently 2–3 years after surgery in portal vein CTC (+) patients (57.1% vs. 8.3%, *P* = 0.038).
Tien *et al*. (2016)	41	SLB μF CMx	At surgery, before any manipulation of cancer	CTCs count in portal venous blood is the significant predictor for liver metastases within 6 months after surgery (*P* = 0.0042).

CTC, circulating tumor cell; *N*, number of patients; SLB, supported lipid bilayer; μF, microfluidic.

### 4.6 Additional markers for CTCs in PDAC

Epithelial–mesenchymal transition (EMT) may explain how the epithelial tumor cells disseminate from primary site and penetrate the endothelium of blood vessel (Chaffer and Weinberg, 2011). Even though the extent of tumor cells undergoing EMT still remains unclear, the epithelial markers (e.g., EpCAM and CK) of epithelial cells are downregulated by EMT‐inducing signals; thus, CTC capture strategies targeting expression of epithelial markers may fail to isolate a subset of CTCs (Krebs *et al*., 2014). The expression of epithelial markers such as EpCAM, CK, and E‐cadherin has been reported to be reduced lower than 40% in CTCs of PDAC (Rhim *et al*., 2012). Similarly, CellSearch^®^ detected CTCs in 39.6% (21/53) of patients with metastatic PDAC, while ISET exhibited better enrichment of CTCs (CTC positivity in 88.9% of patients with metastatic PDAC) (Khoja *et al*., 2012). Combining additional markers for capturing mesenchymal‐like CTCs remain to be identified. Potential mesenchymal markers include the following: ZEB1, SNAI1, vimentin, N‐cadherin, FGFR2, PLS3, Twist1, and PI3K/AKT (Barriere *et al*., 2014). A few recent studies have reported the application of mesenchymal markers to detect CTCs in PDAC. CTCs enriched by ScreenCell^®^ filtration devices were stained with ZEB1 and CK. ZEB1‐positive CTCs were found in almost exclusively in patients with metastatic PDAC (*P *=* *0.01) (Kulemann *et al*., 2016). Dotan *et al*. evaluated 23 patients with metastasis who had at least one CTC detected at baseline by using CellSearch^®^. They assessed for the expression of MUC‐1, which play a role of inducing EMT: MUC‐1 expression was observed in 43% (10/23) of the patients, and patients with CTCs positive for MUC‐1 had shorter median OS than those with CTCs negative for MUC‐1 (2.7 months *vs*. 9.6 months, *P *=* *0.044) (Dotan *et al*., 2016). Another study, which compared epithelial CTCs and mesenchymal‐like CTCs using a vimentin marker, was discussed above (Poruk *et al*., 2016). However, blood cells including monocytes and granulocytes retain vimentin expression during the maturation, which warrant additional confirmation of tumor‐specific markers (Dellagi *et al*., 1983).

A subset of tumor cells, so‐called cancer stem cells (CSCs), have properties of stem cells and display self‐renewing and multipotency capabilities, which are considered to be responsible for metastasis, chemoresistance, and recurrence of tumors (Krebs *et al*., 2014; Satoh *et al*., 2015). It has been reported that CSC and EMT share common molecular pathways (e.g., Wnt/ß‐catenin and Notch signaling), and epithelial cells undergoing EMT acquire CSC features (Igawa *et al*., 2014). Key markers for identifying pancreatic CSCs include CD133 and aldehyde dehydrogenase (ALDH) (Fitzgerald and McCubrey, 2014). Marker combinations of CD44, CD24, and epithelial‐specific antigen (ESA) were also identified as indicators of pancreatic CSCs (Li *et al*., 2007). Other putative markers for pancreatic CSCs include c‐Met, doublecortin‐like kinase 1, and CD44v6 (Polireddy and Chen, 2016). A recent study by *Poruk et al*. evaluated 60 consecutive PDAC patients undergoing surgery. CTCs were detected by IF staining using CK, CD133, CD44, and ALDH, after isolated by ISET. CK+/ALDH+ CTCs and CK+/CD133 + /CD44 + CTCs were detected in 77% (46/60) and in 57% (46/60) of patients, respectively. For the 59 nonmetastatic patients, ALDH‐positive CTCs and CK+/CD133 + /CD44 + CTCs were significantly associated with decreased DFS and higher risk of tumor recurrence (Poruk *et al*., 2017).

## 5 Current technologies in ctDNA

Since ctDNA is present in minute quantity in the bloodstream, extraction of cfDNA without contamination of plasma with genomic DNA is a major challenge in ctDNA analysis. Preanalytical variables that include specimen types (plasma or serum), specimen collection procedures (time to processing of whole blood), blood collection tubes, specimen handling (including centrifugation protocols and temperature), and methods of cfDNA isolation and purification are the most important factors to control this success (Diefenbach *et al*., 2018; Markus *et al*., 2018; Sato *et al*., 2018). Plasma has been preferred as a source for extracting circulating DNA. Even though serum contains 2–24 times higher amount of cfDNA than plasma, serum is not recommended due to the possible contamination from white blood cells during the clotting process (Heitzer *et al*., 2015; Parpart‐Li *et al*., 2017; Trigg *et al*., 2018; Zhao *et al*., 2019). If specimen processing can be performed within 6 h from collection, standard K2EDTA collection tubes are suitable for blood sampling. However, when the processing is delayed by up to 48 h, specialized cell‐stabilizing blood collection tubes should be used to reduce contamination by genomic DNA released from leukocyte lysis (Alidousty *et al*., 2017; Medina Diaz *et al*., 2016; Merker *et al*., 2018; Risberg *et al*., 2018; Ward Gahlawat *et al*., 2019; Warton *et al*., 2017). Current evidence recommends that isolated plasma, not whole blood, can be stored frozen up to 9 months or up to a few years, depending on analytical goals (van Dessel *et al*., 2017; Meddeb *et al*., 2019). The isolated plasma is preferably aliquoted into a single use fraction: A single freeze–thaw cycle had no significant effect on cfDNA stability (Bronkhorst *et al*., 2015; Merker *et al*., 2018). Several issues regarding DNA isolation and nonmalignant conditions that induce the release of cfDNA should be considered, but the following discussion focuses more on the techniques in progress for sensitive detection of the small fraction of ctDNA (Heitzer *et al*., 2015; Qin *et al*., 2016).

Based on PCR technology, new technologies including real‐time quantitative PCR (qPCR) (Brown, 2016), amplification‐refractory mutation system (ARMS)‐based qPCR (Zhang *et al*., 2015c), competitive allele‐specific TaqMan PCR (cast‐PCR) (Ashida *et al*., 2016; Reid *et al*., 2015), coamplification at lower denaturation temperature PCR (COLD‐PCR) (Milbury *et al*., 2011) have been introduced. More recently, digital PCR (dPCR), which uses droplets to compartmentalize individual DNA strands, reached the high sensitivity ranging from 0.1% to 0.001% and is therefore beneficial to detect low allele frequency variants (Gorgannezhad *et al*., 2018; Vogelstein and Kinzler, 1999). dPCR includes droplet PCR, Bio‐Rad droplet dPCR (ddPCR) platform (Hindson *et al*., 2011), and BEAMing (beads, emulsion, amplification and magnetics) (Chen *et al*., 2013): This method is currently among the most promising of targeted approaches, which focuses on the detection of rare mutations in DNA samples with prior knowledge of genetic changes at specific loci of the tumor (e.g., *KRAS, BRCA2, ERBB2,* and *EGFR)* (Alix‐Panabieres and Pantel, 2016; Cheng *et al*., 2017) and exhibits high analytical sensitivity. BEAMing combines emulsion PCR amplification and flow cytometry and therefore can be assessed in the standard laboratory setting (Dressman *et al*., 2003). BEAMing quantifies independently the fluorescently labeled particles, which is able to detect the rare variants with allele frequency < 0.01%. This method enables the counting of error rate of DNA polymerases (Gorgannezhad *et al*., 2018). The ddPCR platform performs PCR amplification within water‐in‐oil emulsion droplets where individual DNA molecules are dispersed in. Using fluorescently labeled probes, droplets can be identified as a binary (mutant‐positive or mutant‐negative) system. The Bio‐Rad QX‐200 platform produces 20 000 droplets and is one of the most commonly used dPCR systems for ctDNA detection (Gorgannezhad *et al*., 2018).

**Figure 1:**
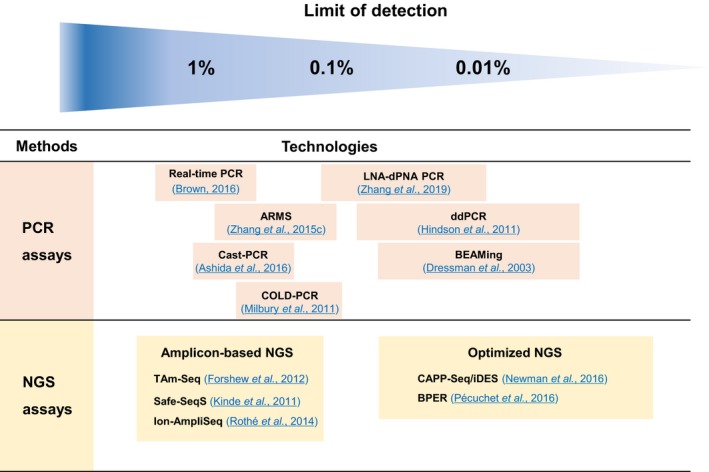
Examples of technology platforms for detecting circulating tumor DNA and limit of detection ranges. These depend on number of mutations measured and quantity of DNA present in a blood sample. Optimized NGS techniques provide sequencing error correction. Other ctDNA assays being applied to pancreatic cancer include personalized panels and commercially available tests. PCR: polymerase chain reaction; NGS: next‐generation sequencing; ARMS: amplification‐refractory mutation system; COLD‐PCR: coamplification at lower denaturation temperature PCR; Cast‐PCR: competitive allele‐specific TaqMan PCR; LNA‐dPNA PCR: locked nucleic acid‐dual peptide nucleic acid PCR clamp; ddPCR: droplet digital PCR; BEAMing: beads, emulsion, amplification, and magnetics digital PCR; TAm‐Seq: tagged‐amplicon deep sequencing; CAPP‐Seq/iDES: cancer personalized profiling by deep sequencing with integrated digital error suppression; BPER: base‐position error rate.

Next‐generation sequencing (NGS), or a massively parallel sequencing, detects a wider range of mutation with higher coverage, but with lower sensitivity (approximately 1%) than dPCR. The targeted NGS approach sequences multiple cancer‐associated genes (Zill *et al*., 2015). Platforms such as safe‐sequencing system (Safe‐SeqS) (Kinde *et al*., 2011), TAm‐Seq (Forshew *et al*., 2012), Ion‐AmpliSeq (Rothe *et al*., 2014), CAPP‐Seq (Newman *et al*., 2014), and sensitive mutation detection using sequencing (SiMSen‐seq) (Stahlberg *et al*., 2017) have been developed. *Zill et al*. used Guardant360 assay to sequence cfDNA in 21 867 advanced cancer patients including 867 PDAC samples and reported the genomic findings and the response outcomes (Zill *et al*., 2018). Recent progress enabled whole‐genome sequencing to be applied to a liquid biopsy (Dawson *et al*., 2013). These NGS approaches largely extended noninvasive profiling of tumors not only focus on single nucleotide variants but also identify structural variants and copy number variations [e.g., personalized analysis of rearranged ends (PARE)] (Leary *et al*., 2012). Recent advances in NGS technology enable similar sensitivity to detection of ctDNA as by digital PCR. A recent study showed a statistical method based on each base‐position error rate (BPER), which detects variants with low allele frequency as low as 0.003 (single nucleotide variation) and 0.001 (insertions/deletions) (Pécuchet *et al*., 2016). Newman *et al*. recently developed an integrated digital error suppression (iDES)‐enhanced CAPP‐Seq, which incorporates in silico removal of artifacts detected in cfDNA sequencing data. This strategy enabled very sensitive detection of tumor‐derived DNA down to 0.002% for generalized iDES‐enhanced CAPP‐Seq and 0.00025% using a customized panel (Newman *et al*., 2016). Other newer methods include the use of bar‐coded amplicon‐based NGS rather than hybrid capture‐based plasma NGS (Guibert *et al*., 2018) or an improved method using dual peptide nucleic acid (PNA) clamping‐mediated locked nucleic acid‐dual peptide nucleic acid PCR clamp (LNA‐dPNA PCR clamp) with sensitivities in the 0.01%‐0.1% range (Zhang *et al*., 2019). Figure 1 summarizes the various technologies and the rages of their limit of detection.

There has been encouraging improvement in the quest for early detection of pancreatic cancer. CancerSEEK, a multi‐analyte blood test, combines multiplex PCR (16 genes) and immunoassay (8 protein biomarkers) (Cohen *et al*., 2018). This method has shown over 69% sensitivity and over 99% specificity for five cancers including pancreatic cancer aiming to screen different cancers in the general population (Kalinich and Haber, 2018).

## 6 Clinical application of ctDNA in PDAC

Previous ctDNA studies in pancreatic cancer are summarized in Table 5. Several studies demonstrated that the amounts of plasma DNA in patients with cancer is higher than those in healthy individuals (Anker *et al*., 1999; Sozzi *et al*., 2003). With regard to PDAC, multiple studies have reported that cfDNA concentration was higher in pancreatic cancer patients compared with normal controls and in advanced stages compared with early stages (Berger *et al*., 2016; Singh *et al*., 2015; Takai *et al*., 2015).

**Table 5:** Previous ctDNA studies focusing on pancreatic cancer

Method	Refs	*N*	Stage	Paired tissue	Sample	Target	Detection rate
ddPCR Bio‐Rad	Bernard *et al*. (2019)	194	R (*n* = 71), M (*n* = 123)	14	Plasma	*KRAS:* ‐ G12D; G12V; G12R; G12C; G12S; G13D	(1) Tissue mutation: 85.7% (12/14), (2) Concordance rate (Tissue vs. ctDNA): 68.2% (15/22), (3) ctDNA: 52.0% (53/102) in R (therapy naïve patients), 31.8% (21/66) in M (therapy naïve patients)
	Kim *et al*. (2018)	106	R (*n* = 41), LA (*n* = 25), M (*n* = 40)	77	Plasma	*KRAS:* G12D; G12V; G12R; G12S; G13D	(1) Tissue mutation: 96.1% (74/77), (2) Concordance rate (Tissue vs. ctDNA): 76.6% (59/77), (3) ctDNA: 77.9% (60/77) in available samples: 68.6% (24/35) in R; 83.3% (5/6) in LA; 86.1% (31/36) in M
	Del Re *et al*. (2017)	27	III (*n* = 4), IV (*n* = 23)	NA	Plasma	*KRAS:* G12D; G12V; G12R; G13D	ctDNA, 70.4% (19/27): 25% (1/4) in stage III; 78% (18/23) in stage IV; G12D 74% (14/19); G12V 11% (2/19); G12R 11% (2/19); G13D 5% (1/19)
	Sefrioui *et al*. (2017)	58^b^	L (*n* = 16), LA (*n* = 18), M (*n* = 24)	27	Plasma	*KRAS*	(1) Tissue mutation: 63% (17/27), (2) Concordance rate (Tissue vs. ctDNA): 70.4% (19/27), (3) cfDNA concentration, median 59.5 ng/mL (range 12.9–925.3 ng/mL): 73.8 ± 45.6 in L; 77.2 ± 41.1 in LA; 122.4 ± 44 in M, (4) ctDNA: 56% (31/55) in available samples, (5) Diagnosis of PDAC using ctDNA: Sensitivity: 65%; Specificity: 75%
	Hadano *et al*. (2016)	105	R (*n* = 105): I (*n* = 2); II (*n* = 82); III (*n* = 3); IV (*n* = 18)	105	Plasma	*KRAS:* G12D; G12V; G12R	(1) Tissue mutation – 82% (86/105): G12D 42% (44/86); G12V 29% (30/86); G12R 11% (12/86). (2) ctDNA – 31% (33/105): G12D 73% (24/33); G12V 21% (7/33); G12R 6% (2/33). (3) ctDNA concentration: mean 10.1 copies of ctDNA/mL
	Berger *et al*. (2016)	87^b^^,^ c	IV	16	Plasmaor Serum	*GNAS:* codon 201; *KRAS;* G12D; G12V	(1) cfDNA concentration: 4.220 ± 2.501 ng·mL^−1^ in PDAC; 0.2887 ± 0.0319 ng·mL^−1^ in IPMN; 0.1360 ± 0.0203 ng·mL^−1^ in NC. (2) *GNAS* ^mut^ ctDNA: 71.4% (15/21) in IPMN; 25.0% (6/24) in PDAC. (3) *KRAS* ^mut^ ctDNA: 41.7% (10/24) in PDAC; 0% in SCA, IPMN and NC. (4) Concordance rate (Tissue vs. ctDNA): 56.3%
	Earl *et al*. (2015)	31	R (*n* = 10), LA (*n* = 8), M (*n* = 13)	12	Plasma	*KRAS:* G12D; G12V; G12R	(1) cfDNA concentration: median 93 RNaseP/20 μL, range 6–1663 RNaseP/20 μL. (2) ctDNA – 26% (8/31): 30% (3/10) in R; 12.5% (1/8) in LA; 30.8% (4/13) in M; G12D (6/8); G12R (1/8); G12V (1/8). (3) Tissue mutation: 58.3% (7/12). (4) ctDNA/Tissue mutation: 60% (3/5)
	Kinugasa *et al*. (2015)	75^b^	II (*n* = 2), III (*n* = 5), IV (*n* = 68)	75	Serum	*KRAS:* G12D; G12V; G12R	(1) Tissue mutation – 74.7% (56/75): G12D 29.3% (22/75); G12V 37.3% (28/75); G12R 8.0% (6/75); (2) ctDNA – 62.6% (47/75): G12D 38.6% (29/75); G12V 34.6% (26/75); G12R 5.3% (4/75). (3) Concordance rate (Tissue vs. ctDNA) – 77.3% (58/75). (4) Specificity – 5% (1/20) in NCs: G12V
	Sausen *et al*. (2015)	51	I (*n* = 2), II (*n* = 45), III (n = 4)	44	Plasma	*KRAS*	(1) ctDNA – 43% (22/51). (2) Specificity: > 99.9%
Chip‐based digital PCR Quanta Studio^®^	Brychta *et al*. (2016)	50^b^	I (*n* = 4), II (*n* = 37), III (*n* = 6), IV (*n* = 3)	50	Plasma	*KRAS:* G12D; G12V; G12C	(1) Tissue mutation – 72% (36/50) for *KRAS* status: G12D 44% (22/50); G12V 20% (10/50); G12C 10% (5/50). (2) ctDNA/Tissue mutation – 35% (13/37): G12D 36% (8/22); G12V 50% (5/10); G12C 0% (0/5). (3) Specificity: 100%
Whole‐exome sequencing Hi‐Seq 2500 ddPCR Bio‐Rad	Cheng *et al*. (2017)	188	M	NA	Plasma	Focused on 60 genes, *KRAS*,* BRCA2, EGFR, KDR,ERBB2*	ctDNA – 83% (156/188): *KRAS* 72.3% (136/188); *BRCA2* 11.7% (22/188); *KDR* 13.8% (26/188); *EGFR* 13.3% (25/188); *ERBB2* exon 17 13.3% (25/188); *ERBB2* exon 27 6.4% (12/188)
Targeted Sequencing Ion PGM™ ddPCR Bio‐Rad	Adamo *et al*. (2017)	26	R (*n* = 6), LA (*n* = 5), M (*n* = 15)	11	Plasma	50 gene panel. *KRAS:* codons 12 and 13	(1) Tissue mutation – 73% (8/11): G12D 50% (4/8); G12V 38% (3/8). (2) cfNDA concentration: 585 ng·mL^−1^ (PDAC); 300 ng·mL^−1^ (CP); 175 ng·mL^−1^ (NC). (3) *KRAS* ^mut^ ctDNA (targeted sequencing, validated by ddPCR) – 26.9% (7/26): 16.7% (1/6) in R; 40% (6/15) in M
Targeted Sequencing Ion Proton™ digital PCR RainDrop™	Pietrasz *et al*. (2017)	135	R (*n* = 31), LA (*n* = 36), M (*n* = 68	NA	Plasma	22 gene panel. *KRAS:* G12D, G12V, G12R	(1) cfDNA concentration: 52.5 ± 79.5 ng·mL^−1^ in R; 105.8 ± 227.25 ng·mL^−1^ in LA‐M. (2) ctDNA – 48% (50/104) in LA‐M: *KRAS* (*n* = 43); *TP53* (*n* = 23); *SMAD4* (*n* = 8); *NRAS* (*n* = 2); *PIK3CA* (*n* = 1); *STK11* (*n* = 1)
Targeted Sequencing MiSeq ddPCR Bio‐Rad	Berger *et al*. (2018)	20	IV	11	Plasma	7 gene panel. *KRAS, TP53*	(1) Tissue mutation: 63.6% (7/11) for KRAS status. (2) ctDNA: 100% (11/11) in therapy naïve patients; 55.6% (5/9) in pretreated patients
digital PCR RainDrop™ Targeted Sequencing Ion Proton™	Pecuchet *et al*. (2016)	100^b^	R (*n* = 23), LA‐M (*n* = 77)	NA	Plasma	*KRAS, EGFR,* 22 gene panel	(1) Amplicon Sequencing (digital PCR as a reference method)^a^: Sensitivity 97.6%; Specificity 94.0%; Accuracy 97.4%. (2) Method comparison (Amplicon Sequencing vs. digital PCR): Highly correlated mutation AF (*R* ^2^ = 0.95)
digital PCR RainDrop™ Targeted Sequencing HiSeq 2500 Ion PGM™	Takai *et al*. (2016); Takai *et al*. (2015)	259	IA (*n* = 3), IB (*n* = 2), IIA (*n* = 29), IIB (*n* = 44), III (*n* = 17), IV (*n* = 163), NA (*n* = 1)	NA	Plasma	*KRAS:* G12D; G12V; G12R; G13D; 60 gene panel	(1) ctDNA (digital PCR based screening): 32% (83/259). (2) ctDNA (confirmed by targeted sequencing): 93.7% (45/48); 93.3% (42/45) was detected by digital PCR as well
Targeted Sequencing HiSeq 2500	Pishvaian *et al*. (2017)	34	NA	23	Plasma	68 gene panel	(1) Tissue mutation: 87% (20/23) for *KRAS* status. (2) ctDNA – 56% (19/34): mutations in median 2 genes/patient; 29% (10/34) for *KRAS* status. (3) Concordance rate (Tissue vs. ctDNA): 39% (9/23) for *KRAS* status
	Zill *et al*. (2015)	26^b^	III (*n* = 3), IV (*n* = 23)	26	Plasma	54 gene panel	ctDNA/Tissue mutation: sensitivity 92.3%; specificity 100%; accuracy 97.7%
Targeted Sequencing MiSeq HiSeq 4000	Cohen *et al*. (2017)	221	R	152 (*TP53*)50 (*KRAS*)	Plasma	*KRAS, TP53* (*n* = 152)	(1) Tissue mutation: 100% (50/50) for *KRAS* status; 42% (64/152) for *TP53* status. (2) ctDNA – *KRAS* 30% (66/221) in plasma: 94% (62/66) in codon 12; 6% (4/66) in codon 61; (3) ctDNA/Tissue mutation: *TP53* 20% (13/64) in paired plasma
Targeted Sequencing Ion PGM™	Perets *et al*. (2018)	17	M	NA	Plasma	*KRAS:* exon 2	ctDNA: 29.4% (5/17)
	Calvez‐Kelm *et al*. (2016)	437	L (*n* = 39), Reg (*n* = 143), Sys (*n* = 135), NA (*n* = 120)	NA	Plasma	*KRAS:* codons 4–16, 51–69	ctDNA – 21.1% (92/437) in total: 10.3% (4/39) in L; 17.5% (25/143) in Reg; 33.3% (45/135) in Sys
CancerSEEK	Cohen *et al*. (2018)	1005^a^	I‐III	NA	Plasma	16 gene panel, 8 proteins	ctDNA: Sensitivities ranged from 69 to 98% for detection of five cancer types including pancreatic cancer; Specificity > 99%
BEAMing (*n* = 2) PCR ligation (*n* = 50) Safe‐SeqS (*n* = 103)	Bettegowda *et al*. (2014)	155	I (*n* = 22), II (*n* = 94), III (*n* = 5), IV (*n* = 34)	155	Plasma	*KRAS:* codons 12,13, 59, 60 and 61	ctDNA – 57.4% (89/155) in total: 48.8% (59/121) in Stage I‐III; 88.2% (30/34) in Stage IV
PNA‐mediated real‐time PCR clamping	Tjensvoll *et al*. (2016)	14^b^	LA (*n* = 2), M (*n* = 12)	NA	Plasma	*KRAS*	ctDNA: 71% (10/14)
	Dabritz *et al*. (2009)	56^b^	Inop (*n* = 23), Op (*n* = 25), NA (*n* = 8)	NA	Plasma	*KRAS*	ctDNA: 36% (20/56)
Microarray‐mediated methylation assay MethDet56	Liggett *et al*. (2010)	30^b^	NA	NA	Plasma	Methylation	Differentiate PC from CP: Sensitivity 91.2%; Specificity 90.8%
	Melnikov *et al*. (2009)	34	R (*n* = 25), NR (*n* = 9)	NA	Plasma	Methylation	Differentiate PC from NC: Sensitivity 76%; Specificity 59%
MSP Nested PCR Direct sequencing	Jiao *et al*. (2007)	83	L (*n* = 16), LA (*n* = 37), M (*n* = 30)	9	Plasma	Methylation: *p16;* ‐ *ppENK; KRAS;* codon 12	ctDNA – 62.6% (52/83) with ≥ 1 alteration: *KRAS* 32.5% (25/77); *ppENK* 29.3% (22/75); *p16* 24.6% (14/57)
Nested PCR	Singh *et al*. (2015)	127^b^^,^ d	No M (*n* = 74), M (*n* = 53)	NA	Plasma	*KRAS:* codon 12	(1) cfDNA concentration: mean 85.2 ± 49.1 ng·mL^−1^ in patients; mean 35.4 ± 7.4 ng·mL^−1^ in NC. (2) ctDNA – 30.9% (34/110) in available samples: GAT 55.9% (19/34); TGT 17.6% (6/34); CGT 26.5% (9/34)
COLD‐PCR combined with unlabeled‐probe HRM approach	Wu *et al*. (2014)	36	NA	36	Plasma	*KRAS:* codon 12, 13	ctDNA – 72.2% (26/36): All of 26 tissue DNA were *KRAS* ^mut^.
Colorimetric‐based assay STA™	Ollar *et al*. (2010)	14	NA	14	Peripheral blood	*KRAS:* codon 12 (GGT>TGT)	ctDNA/Tissue mutation: 21.4% (3/14): Tissue (+), PB (−); 7.1% (1/14): Tissue (−), PB (+); 71.4% (10/14): Tissue (−), PB (−)
MLA	Uemura *et al*. (2004)	28^b^	I (*n* = 2), II (*n* = 8), III (*n* = 7), IVA (*n* = 7), IVB (*n* = 4)	28	Plasma	*KRAS:* exon 1	(1) ctDNA/Tissue mutation: 93% (26/28) in tissue; 35% (9/26) in paired plasma. (2) Specificity – No mutation in normal DNA
Direct sequencing	Chen *et al*. (2010)	91	III (*n* = 29), IV (*n* = 62)	NA	Plasma	*KRAS:* codon 12	ctDNA – 33% (30/91): G12D 56.7% (17/30); G12V 36.7% (11/30); G12R 6.7% (2/30); 17.2% (5/29) in Stage III; 40.3% (25/62) in Stage IV
PCR‐RFLP	Dianxu *et al*. (2002)	41	I (*n* = 2), II (*n* = 6), III (*n* = 5), IV (*n* = 26), NA (*n* = 2)	36	Plasma	*KRAS:* codon 12	(1) Tissue mutation: 91.7% (33/36). (2) ctDNA – 70.7% (29/41); (3) ctDNA/Tissue mutation – 75.8% (25/33) in paired plasma. (4) Specificity – 100% (3/3): Tissue (−) and ctDNA (−)
PCR‐RFLP Direct sequencing	Mulcahy *et al*. (1998)	21	NR	10	Plasma	*KRAS:* codon 12	ctDNA – 81% (17/21): before clinical diagnosis in 4 patients

AF, allele frequency; cfDNA, cell‐free DNA; CP, chronic pancreatitis; ctDNA, circulating tumor DNA; ddPCR, droplet digital PCR; Inop, inoperable; IPMN, intraductal papillary mucinous neoplasm; L, local; LA, locally advanced; M, metastatic; MLA, mismatch ligation assay; MSP, methylation‐specific PCR; *N*, number of patients; NA, not available; NC, normal control; NR, nonresectable; Op, operable; PC, pancreatic cancer; PDAC, pancreatic ductal adenocarcinoma; PNA, peptide nucleic acid; R, resectable; Refs, references; Reg, regional; Safe‐SeqS, Safe‐Sequencing System; SCA, serous cystadenoma; Sys, systematic.

a Results from other types of cancer patients are included.

b Various tumor types of pancreatic cancers are included.

c Five study cohorts, PDAC (*n* = 24); IPMN (*n* = 21); borderline IPMN (*n* = 16); SCA (*n* = 26)

d *KRAS* mutation test was available for 110 samples.

### 6.1 Method comparison

Pécuchet *et al*. evaluated 77 patients with pancreatic cancer and compared a microfluidic dPCR (RainDrop^®^) and NGS analysis (Ion Proton™) in detecting *KRAS* and *EGFR* mutations. 97.4% (75/77) of results were concordant. *KRAS* mutation was only detected by dPCR in two samples (allele frequency 0.003 and 0.006, respectively) (Pécuchet *et al*., 2016). Similarly, Pietrasz *et al*. assessed 135 patients with PDAC and compared the two methods in detecting *KRAS* mutant ctDNA. They reported high concordance (*R* ^2^ = 0.94) between the targeted NGS analysis (Ion Proton™) and dPCR (RainDrop^®^) in detecting *KRAS* mutant ctDNA: One sample considered as *KRAS* mutation‐negative in NGS analysis was positive in dPCR (allele frequency 0.0061) (Pietrasz *et al*., 2017). Takai *et al*. applied a two‐stage strategy to analyze *KRAS* mutant ctDNA in PDAC patients. They used ddPCR (Bio‐Rad) as a prescreening method and then performed NGS analysis (Illumina HiSeq 2000) for 60 genes including *KRAS* (Takai *et al*., 2015). The use of NGS analysis as a prescreening method, combined with ddPCR (Bio‐Rad) for further validation, has also been successfully applied for ctDNA analysis (Adamo *et al*., 2017; Cheng *et al*., 2017). The combined strategy was suggested as cost‐effective and efficient method for analyzing ctDNA in PDAC patients (Takai *et al*., 2015). Further approaches to establish efficient strategies for analyzing tumor genomes in plasma DNA are highly warranted.

### 6.2 Early diagnosis

According to the recent joint review by the American Society of Clinical Oncology (ASCO) and the College of American Pathologists (CAP), further studies are still required to prove the clinical utility of ctDNA in early diagnosis (Merker *et al*., 2018). IPMNs are the most frequent potentially malignant pancreatic cysts and classified into main duct type (MD‐IPMN) and branch duct type (BD‐IPMN). Since only 15–20% of BD‐IPMN will develop malignancy and nonsurgical management is recommended for low‐risk BD‐IPMNs, we need to correctly identify malignant IPMNs. Recently, an imaging tool that is combined with the identification of genomic patterns, coined ‘radiomics’, has been proposed by several studies (Hanania *et al*., 2016; Permuth *et al*., 2016). Similarly, *Berger et al*. detected *GNAS* mutant plasma DNA in 71.4% (15/21) of IPMN patients, but neither in serous cyst adenoma patients nor in healthy controls. ctDNA assay can be a useful tool for the discrimination of IPMN with malignant potential from other harmless pancreatic tumors, even though additional approaches to differentiate low from high‐grade IPMN is still required (Berger *et al*., 2016).

For realizing early cancer detection using ctDNA‐based screening tests, an interesting clinical trial (https://clinicaltrials.gov/ct2/show/NCT02889978) by a company (GRAIL, Inc) is currently ongoing and recruiting 15 000 participants including cancer subjects with multiple types and healthy subjects. This project, called the Circulating Cell Free Genome Atlas (CCGA), aims to identify potential cancer mutations and to complete a reference database of the mutations in circulating DNA in plasma (Aravanis *et al*., 2017).

### 6.3 Prognostic marker

Previous ctDNA studies mostly focused on *KRAS* hotspot (codon 12) mutations and its association with clinical outcomes of patients with PDAC. Sausen *et al*. (2015) demonstrated that patients with *KRAS* mutant ctDNA after surgery were more likely to relapse than those without *KRAS* mutant ctDNA (9.9 months *vs*. not reached, *P *=* *0.02). Another study evaluated PDAC patients undergoing surgery and reported that the detection of ctDNA by ddPCR at baseline correlated with shorter DFS and OS (DFS, 6.1 months *vs*. 16.1 months; OS, 13.6 months *vs*. 27.6 months; *P *<* *0.001 and *P *<* *0.0001, respectively) (Hadano *et al*., 2016). This was also confirmed by Earl *et al*. (2015) in which patients with ctDNA detected by ddPCR had significantly shorter OS than patients with no detectable ctDNA. In metastatic PDAC, undetectable *KRAS* mutant ctDNA was significantly associated with survival benefit (8 months *vs*. 37.5 months, *P *<* *0.004) (Perets *et al*., 2018). For patients with resectable disease, MST of patients in whom ctDNA was detected were significantly shorter than those of patients in whom ctDNA was not detected (3.9 months *vs*. 10.2 months, *P *<* *0.001) (Chen *et al*., 2010). Furthermore, it has been reported that high amount of cfDNA is a relevant prognostic marker for pancreatic cancer patients (Singh *et al*., 2015; Tjensvoll *et al*., 2016). A recent meta‐analysis by Creemers *et al*. (2017) showed that the ctDNA in pancreatic cancer is significantly associated with a poor prognosis. In contrast, Bernard *et al*. (2019) analyzed longitudinal *KRAS* mutant allele fraction from ctDNA and exosome DNA and determined that longitudinal monitoring through exosome DNA rather than ctDNA provides prognostic information.

### 6.4 Predictive marker

So far, the role of ctDNA as a relevant predictive marker in PDAC remains to be identified. Recently, reported predictive markers for gemcitabine response are limited to the germline variants (Innocenti *et al*., 2012; Li *et al*., 2016). In a phase III trial, comparing gemcitabine alone with erlotinib plus gemcitabine, the OS was significantly prolonged on the combined therapy, yet *EGFR* status did not predict the response to the therapy (Moore *et al*., 2007). As the frequency of *KRAS* mutation in PDAC ranges from 88 to 100%, current efforts are underway to target *KRAS* pathway to make therapeutic progress in PDAC (Collisson *et al*., 2012; Krantz and O'Reilly, 2018; Rao *et al*., 2004; Van Cutsem *et al*., 2004; Ying *et al*., 2016). Additionally, targeting pancreatic CSCs, γ‐secretase inhibitors (GSI) to inhibit Notch signaling pathway have been developed (Abel *et al*., 2014; Whitehead *et al*., 2012). With regard to the epigenetic regulation, deregulation of histone deacetylases (HDACs) has been reported to play a role in pancreatic cancer development (Polireddy and Chen, 2016). HDAC inhibitors are currently tested for pancreatic cancer treatment, but there seems to be no benefit in clinical outcomes (Millward *et al*., 2012; Richards *et al*., 2006; Tinari *et al*., 2012). In this context, ctDNA assay will have clinical utility in noninvasive molecular profiling for the novel druggable mutations. An NGS approach targeting 60 cancer‐associated genes identified potentially targetable mutations in plasma DNA of PDAC patients (Takai *et al*., 2015).

## 7 Future perspectives

Early detection, real‐time disease monitoring, molecular profiling for targeted therapy are applications that promise to improve pancreatic cancer management. Liquid biopsy is a potentially valuable tool for in this regard. Multiple studies revealed the clinical use of liquid biopsy in monitoring patients (Table 6). ctDNA analysis may be more sensitive, easily accessible, and suitable not only for monitoring tumor dynamics during treatment, but for noninvasive molecular profiling of tumors due to the high incidence of nongermline (as well as some germline) genetic variations (Cicenas *et al*., 2017). Several recent studies have performed ctDNA analysis targeting noncoding repetitive DNA sequence such as *ALU* and described the possible use of noncoding DNA as additional prognostic marker in cancer monitoring (Chang *et al*., 2017; Lehner *et al*., 2013). CTC analysis, however, has its own strengths in that CTCs enable functional analyses such as drug testing, particularly as they represent cells still remaining after previous treatment during the course of disease. Thus, we suggest that both CTCs and ctDNA can be used in future parallel or complementary analyses (Kidess‐Sigal *et al*., 2016) and it is hoped that both these technologies will influence future diagnosis and treatment of this currently devastating disease. In addition to CTCs and ctDNA, there is increasing attention for emerging role of extracellular vesicles (EVs). Exosomes are a well‐studied EV population and can be a source for tumor‐specific proteins and RNAs (i.e., mRNA, noncoding RNA, and miRNA). Exosomes that carry cargo consisting of disease‐specific nucleic acids and proteins can provide a promising tool for characterizing cancer specific features as well as targeted treatment in pancreatic cancer (Kamerkar *et al*., 2017; Massoumi *et al*., 2019; Qian *et al*., 2019; Qiu *et al*., 2018; Siravegna *et al*., 2017).

**Table 6:** Studies that revealed the clinical use of CTCs/ctDNA in monitoring patients

Reference	Analyte	Time point measuring CTCs/ctDNA	Results
Dotan *et al*. (2016)	CTCs	First disease evaluation (6–10 weeks after treatment initiation)	For patients with ≥ 1 CTCs at diagnosis, 47% (7/15 patients) had no CTCs detected at first disease evaluation.
Sheng *et al*. (2014)	CTCs	First day of each subsequent treatment cycle.	The CTC number correlated proportionally with CT scan measured tumor size in each of the three patients.
Bernard *et al*. (2019)	ctDNA	Baseline Immediately after neoadjuvant therapy completion (*n* = 34) in resectable PDAC At least two consecutive samples within the same treatment regimen (*n* = 34) in metastatic PDAC	Reduction in ctDNA after completion of neoadjuvant therapy did not correlate with progression (resectable PDAC). Reduction in exoDNA MAF after completion of neoadjuvant therapy correlated with progression (OR = 38.4; *P* = 0.0002) (resectable PDAC). Serial ctDNA MAF did not correlate with progression in metastatic PDAC. Any on‐treatment serial exoDNA sample was significantly associated with eventual progression (*P* < 0.0001) in metastatic PDAC.
Berger *et al*. (2018)	ctDNA	Baseline 4 weeks after treatment at disease progression	The median CMAF level significantly decreased during treatment (*P *=* *0.0027) and increased during progression (*P *=* *0.0104). CMAF levels during treatment significantly correlated with PFS (*P *=* *0.0013 )
Del Re *et al*. (2017)	ctDNA	Subsequently after 15 days of Tx and at first radiologic evaluation	*KRAS* ^mut^ ctDNA change (at the 15‐day sample) correlated with PFS (increase, 2.5 months vs. stability/reduction, 7.5 months; *P *=* *0.03). *KRAS* ^mut^ ctDNA change (at the time of first radiologic evaluation) correlated with PFS (increase, 2.8 months vs. reduction, 7.5 months; *P *=* *0.028).
Tjensvoll *et al*. (2016)	ctDNA	Subsequently every month during treatment	ctDNA measurements could reveal disease progression at an earlier stage for some patients compared to conventional monitoring methods.
Sausen *et al*. (2015)	ctDNA	Multiple time points after surgery	Patients with detectable ctDNA after surgical resection were more likely to relapse than those with undetectable alterations (*P *=* *0.02)

CMAF, combined mutational allele frequency; CT, computed tomography; CTC, circulating tumor cell; ctDNA, circulating tumor DNA; exoDNA, exosome DNA; MAF, mutant allele fraction; PFS, progression‐free survival.

At this time, however, based on an extensive joint review on ctDNA by the American Society of Clinical Oncology and the College of American Pathologist, there are still many questions regarding the clinical validity and clinical utility of ctDNA assays in cancer screening, early‐stage disease, and treatment monitoring (Merker *et al*., 2018). Further research, development of tools utilizing ctDNA, and clinical practice guidance are warranted.

The liquid biopsy field will require further investigation with particular emphasis on clinical utility, not only clinical validation. This means demonstrating that liquid biopsy assay results will affect patient care in specific ways (e.g., changes in surgical approach and/or use of neoadjuvant therapies, changes in drug treatments) and that such changes will improve the morbidity and mortality of pancreatic cancer that we hope will occur in the near future. In particular, prospective clinical trials will be required that show that ctDNA may predict which patients are more likely to respond to chemotherapy and/or immune therapy and/or radiation therapy, or whether specific genetic aberrations identified in blood products (CTCs, ctDNA, EVs) predict response to particular therapies. For example, an exosomes test from Exosome Diagnostics is being tested as a companion diagnostic for Intezyne's phase 1/2 clinical trials of IT‐139, a novel cancer resistance pathway (CRP) inhibitor for the treatment of pancreatic, gastric, and other cancers in combination with existing anticancer therapies. Equally exciting it that the potential use of targeted EVs as a systemic treatment. A phase I trial that studies the best dose and side effects of mesenchymal stromal cells‐derived exosomes with *KRAS* ^G12D^ siRNA (iExosomes) has been approved by the U.S. National Cancer Institute (https://clinicaltrials.gov/ct2/show/NCT03608631) but not yet started. It will be used for the treatment of participants with metastatic pancreatic cancer with *KRAS* ^G12D^ mutation, hoping that iExosomes may prove a better treatment for this dismal disease.

In summary, while liquid biopsy is promising, it still remains a burgeoning field. However, there are many positive signs that it will have a strong impact on the diagnosis, monitoring, and treatment of pancreatic cancer.

## Conflict of interest

The authors declare no conflict of interest.

## Author contributions

J‐SL and SSJ wrote the manuscript and created the figure and graphical abstract. SSP, YKL, and JAN edited the manuscript. All authors reviewed and approved the final version.
